# Network Pharmacology-Based Approach to Investigate the Molecular Targets of Rhubarb for Treating Cancer

**DOI:** 10.1155/2021/9945633

**Published:** 2021-06-08

**Authors:** Lan Jiang, Zhongquan Shi, Yi Yang

**Affiliations:** ^1^Chongqing Three Gorges Medical College, Chongqing 404120, China; ^2^Chongqing Engineering Research Center of Antitumor Natural Drugs, Chongqing 404120, China; ^3^Chongqing University Three Gorges Hospital, Chongqing 404000, China

## Abstract

**Background:**

As a traditional Chinese medicine, rhubarb (also named Dahuang) is used to treat various diseases.

**Objective:**

To explore the possible antitumor mechanism of rhubarb by using network pharmacology and molecular docking in this study.

**Methods:**

Bioactive ingredients and related targets of rhubarb were obtained from the Traditional Chinese Medicine Systems Pharmacology (TCMSP) database. And the gene names corresponding to the proteins were found in the UniProt database. Then, the tumor-related targets were screened out from GeneCards and OMIM databases. Key antitumor targets of rhubarb were acquired by overlapping the above targets via the Venn diagram. The antitumor targets network of rhubarb active components was constructed by using Cytoscape 3.6.0 software. The protein interactions network was constructed using the STRING database. The GO and KEGG pathways involved in the targets were analyzed by using the DAVID database. Autodock Vina software was used to verify the molecular docking of rhubarb components and key targets.

**Results:**

Through screening and analysis, 10 active ingredients and 58 antitumor prediction targets were obtained and constructed a compound-target network. The targets such as CASP3, JUN, MYC, TNF, and PTGS2 may play a crucial role. These targets are involved in cancer pathway, calcium signaling pathway, cell apoptosis, small-cell lung cancer pathway, p53 signaling pathway, and TNF signaling pathway. The docking results indicated that the rhein binding with the CASP3 showed the highest binding energy.

**Conclusion:**

Based on the network pharmacology, the characteristics of multicomponent, multitarget, and multipathway of rhubarb were discussed, which provided a scientific basis for explaining the mechanism in treating cancer and new ideas for further research.

## 1. Introduction

Rhubarb (also named Dahuang), one of the most ancient herbs, belongs to the *Rheum L.* genus of the Polygonaceae family. Rhubarb has long been used as an antibacterial, anti-inflammatory, antifibrotic, and anticancer medicine in China [1, 2]. It contains various active components, including anthraquinones, anthrone, acetophenone, flavonoids, and polysaccharides [3–7]. In recent years, there have been increasing reports about rhubarb's antitumor effects. It has been found that rhubarb has inhibitory effects on liver cancer, breast cancer, gastric cancer, and other tumors [8–12]. However, as one traditional Chinese medicine, the composition of rhubarb is complex and the mechanism of molecular action is still unclear. Therefore, with the help of network pharmacology methods, it is meaningful to explore the relevant targets and mechanisms of its antitumor effect.

Network pharmacology is based on systems biology to construct a biological network, reveal the pharmacological effects of drugs, and explore the relationship between drugs and diseases from a macro perspective. It is especially suitable for analyzing the pharmacodynamic mechanism of traditional Chinese medicines with more complex ingredients [13–16]. Molecular docking is a computational method that predicts the binding interaction of the small-molecule ligands and the target proteins, and it can be applied to confirm and check the result of network pharmacology.

In this study, network pharmacology and molecular docking methods were used to analyze the antitumor pharmacological effects of rhubarb, which may provide a basis for further experimental investigation.

## 2. Materials and Methods

### 2.1. Screening Active Components of Rhubarb

The Chinese Medicine System Pharmacology Database (TCMSP, (http://tcmspw.com/tcmspsearch.php) is an open and accessible database resource containing information about active compounds, potential targets, and related diseases of Chinese medicine. The keyword “Dahuang” was entered into the search box. The effective compounds were screened by pharmacokinetic absorption, distribution, metabolism, and excretion (ADME) criteria: oral bioavailability (OB) ≥ 30% and drug-likeness (DL) ≥ 0.18 [17–19].

### 2.2. Predicting the Targets of the Compounds

Prediction of possible targets related to rhubarb compounds was performed by searching “Related Targets” of the TCMSP Database. And the gene names corresponding to the proteins were found in UniProt (https://www.uniprot.org/) [20]. The network analysis software Cytoscape 3.6.0 was used to construct a network of antitumor active ingredients of rhubarb-predicting target points and analyze the network structure, where each node in the network represented a gene or molecule, and the connection between two nodes represented the connection between the existing connections, the greater the degree of the node (degree), the greater the role of the target in the network.

### 2.3. Screening of Gene Targets

The tumor-related genes were collected from GeneCards (http://www.genecards.org) and OMIM (https://omim.org/) with “tumor” as the keyword. The targets corresponding to rhubarb active components and the targets retrieved from the disease database were crossed, and the repeated targets were deleted to obtain the antitumor targets of rhubarb.

### 2.4. Construction and Analysis of Protein-Protein Interaction Network

The STRING database (https://string-db.org/) is an online protein interaction (PPI) analysis database. It can use computational predictions to supplement the existing information on protein-protein interactions [21, 22]. The 58 antitumor targets of rhubarb were uploaded to the STRING database. The species was set as *Homo sapiens*, and the minimum interaction score was 0.4 to build a protein interaction network. The results were exported as a “TSV” format file and imported into the Cytoscape 3.6.0 version for visual analysis. Set the size and color of the node according to the degree value, and set the thickness of the edge according to the combination score.

### 2.5. GO (Gene Ontology) and KEGG Pathway Enrichment

GO (Gene Ontology) is a database established by the Gene Ontology Federation. There are three categories of GO databases, namely, cellular component (CC), biological process (BP), and molecular function (MF), which describe the cellular environment in which the gene product is located, the biological processes involved, and the possible molecular functions, respectively. The Kyoto Encyclopedia of Genes and Genomes (KEGG) pathway is a knowledge base including most of the known metabolic pathways and some of the known regulatory pathways [23].

The DAVID online database (https://david.ncifcrf.gov/) is an online biological knowledge base and an analytic tool to extract biological information about gene functional classification, functional annotation, and enriched pathways. The targets of rhubarb were imported into the database, and the species was defined as *Homo sapiens* for GO and KEGG enrichment analysis. *P* < 0.05

was used for GO enrichment analysis; and *P* < 0.01

was used for KEGG enrichment analysis. A bubble chart was plotted using the OmicShare platform, a free online platform for data analysis (http://www.omicshare.com/tools).

### 2.6. Molecular Docking of Active Components with Key Targets

Select the target with the largest value in the PPI network as the receptor and the active ingredients of rhubarb having the number of gene targets >3 as the ligand for molecular docking verification. The method was used as follows: First, the two-dimensional (2D) structure diagrams of these compounds were obtained from the PubChem database (https://pubchem.ncbi.nlm.nih.gov) and imported into Chem3D software to draw three-dimensional (3D) structure diagrams and optimize energy and save them in mol2 format. Then, the files were imported into AutoDockTools-1.5.6 software and saved in pdbqt format. Next, the protein crystal structures corresponding to the core target genes were obtained from the PDB database (https://www.rcsb.org/), imported into PyMOL software to remove water molecules and heteromolecules, then imported into AutoDockTools-1.5.6 software to add hydrogen atoms, saved in pdbqt format, and searched their active pockets. Finally, the compound was used as a ligand and the protein as a receptor for molecular docking. PyMOL software was used to analyze and interpret the results [24, 25]. The docking effects were evaluated by the affinity value. The affinity values ≤−9, ≤−7, and≤−5 kcal/mol represent strong, good, and certain binding activity, respectively.

## 3. Results

### 3.1. Screening Active Components of Rhubarb

In the TCMSP database, “Dahuang” was used as the keyword to search chemical components, and 92 chemical components were obtained. The parameters were set as follows: oral bioavailability (OB ≥ 30%), drug-like properties (DL ≥ 0.18), and components without corresponding targets were screened out. Finally, a total of 10 potential effective components were obtained (Table 1).

### 3.2. Collection of Target Information and Construction of Active Component-Target Network

**Figure 1:**
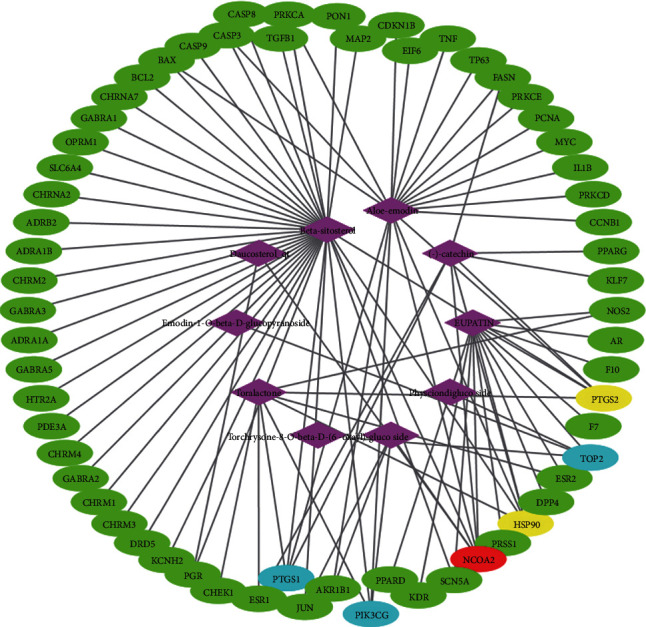
Effective component-target network of Chinese rhubarb.

The collected 10 effective chemical components of rhubarb were searched on the TCMSP platform for the corresponding target protein, and the gene names corresponding to the proteins were found in UniProt. The network analysis software Cytoscape 3.6.0 was used to construct the antitumor active ingredient-target network. The result is shown in Figure 1. The network had 72 nodes and 95 edges. The purple diamond node represented the active ingredients of rhubarb, and the oval node represented the target genes. If the target had 6 active ingredients, it was marked in red. If the target had 5 active ingredients, the corresponding component was marked with yellow; if the target had 4 active ingredients corresponding to it, it was marked with blue; and if the target had only 1–2 active ingredients corresponding to it, it was marked with green. It can be drawn from the figure that almost every active ingredient of rhubarb had multiple targets, and a target can also be related to multiple active ingredients, reflecting the characteristics of multicomponent and multitarget action of rhubarb.

The target number of the 10 active ingredients from compound NO.1 to NO.10 was 14, 1, 4, 1, 8, 1, 2, 36, 20, and 8, respectively.

### 3.3. Screening of Gene Targets

**Figure 2:**
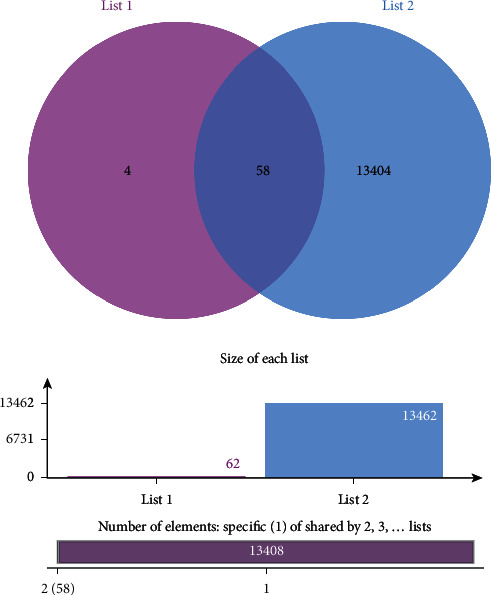
Venn map of drug and disease targets. List 1 (rhubarb-related targets) and List 2 (cancer-related targets).

The keyword “tumor” was used to search the reported tumor-related genes in the two disease databases OMIM database and GeneCards database. A total of 13462 genes were obtained after deletion of duplicates. Key antitumor targets of rhubarb were acquired by overlapping the above targets via the Venn diagram. A total of 58 antitumor targets of rhubarb were obtained. The results are shown in Figure 2.

### 3.4. Construction and Analysis of Protein-Protein Interaction Network

**Figure 3:**
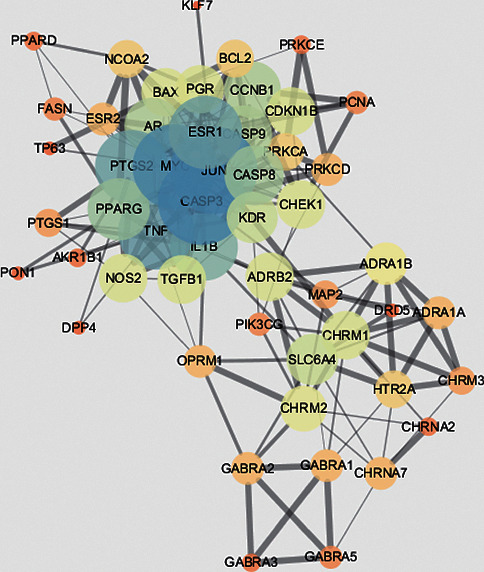
Interaction network of targets for Chinese rhubarb on cancer.

The 58 antitumor targets of rhubarb were entered into the STRING database, the PPI results were finally obtained, and the results were drawn using the network analysis software Cytoscape 3.6.0. (see Figure 3). The network involved 52 nodes and 229 edges. The greater the degree of the node, the greater the role of the target in the network. In the network, CASP3 (24), JUN (24), MYC (23), TNF (20), and PTGS2 (19) were the top 5 nodes in terms of degree value. It was speculated that these nodes may be the key antitumor targets of rhubarb.

### 3.5. GO (Gene Ontology) and KEGG Pathway Enrichment

**Figure 4:**
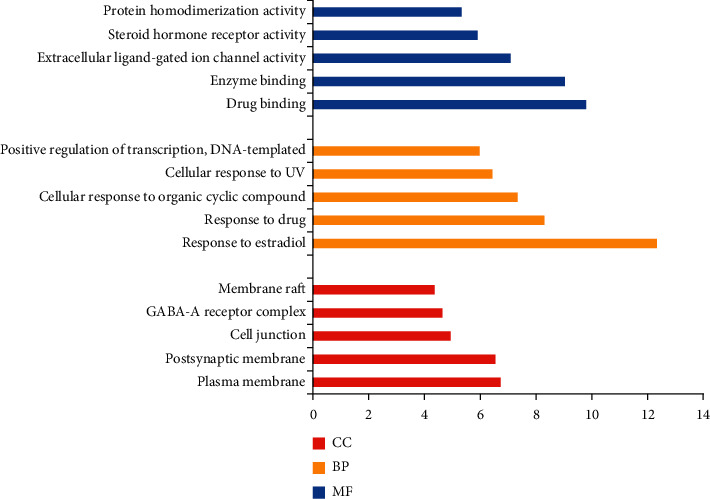
GO (Gene Ontology) biological function analysis results.

To further analyze its target genes, the target of rhubarb was entered into the DAVID database for GO analysis and KEGG pathway analysis. The results showed that the predicted target genes of rhubarb were mainly enriched in 191 biological processes (BP), 27 cellular components (CC), and 62 molecular functions (MF). The top 5 enriched conditions of GO analysis are shown in Figure 4. Red represents CC, orange represents BP, and blue represents MF. Cell components mainly included plasma membrane, postsynaptic membrane, cell junction, GABA-A receptor complex, and membrane raft. Biological process mainly included the response to estradiol, response to drug, cellular response to organic cyclic compound, cellular response to UV, positive regulation of transcription, and DNA-templated. Molecular functional targets mainly included drug binding, enzyme binding, extracellular ligand-gated ion channel activity, steroid hormone receptor activity, and protein homodimerization activity.

**Figure 5:**
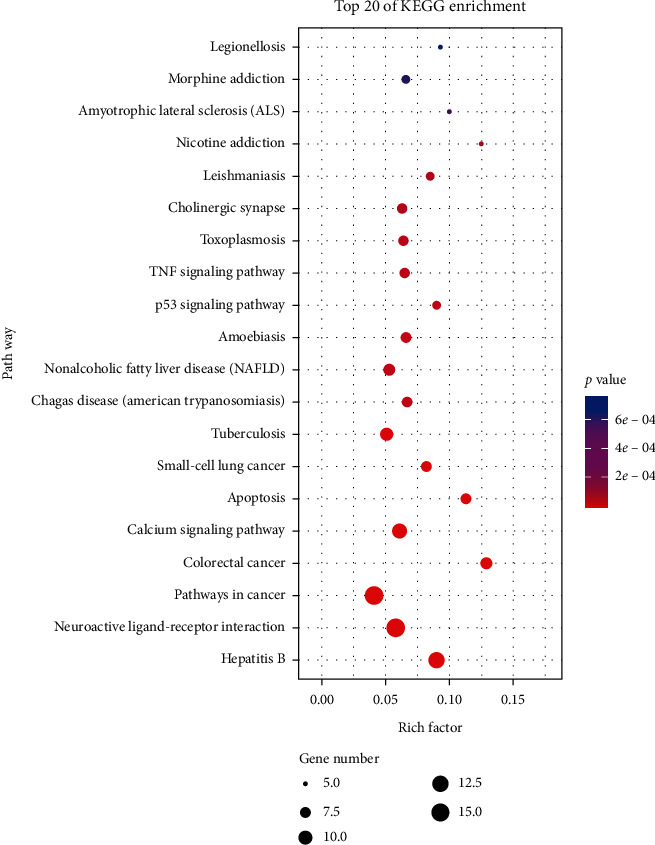
KEGG pathway analysis results of intersection genes (Top 20).

The KEGG pathway enrichment has obtained a total of 75 enrichment pathways, of which the first 20 pathways are shown in Figure 5. The *Y*-axis represents the name of the pathway, the *X*-axis represents the ratio of targeted genes to background genes, the size of the dot represents the number of genes concentrated on the modified pathway, and the color of the dot represents the significance of enrichment. KEGG pathway analysis targets mainly involve hepatitis B pathway, cancer pathway, calcium signaling pathway, apoptosis, small-cell lung cancer pathway, p53 signaling pathway, and tumor necrosis factor signaling pathway, indicating that rhubarb may act on these signaling pathways in the treatment of tumors.

### 3.6. Molecular Docking of Active Components with Key Targets

In this study, CASP3 and JUN with the highest median value in the PPI network were selected as the protein receptors. The active ingredients including EUPATIN, rhein, toralactone, beta-sitosterol, aloe-emodin, and (−)-catechin, which have the number of gene targets >3, were used as ligands for molecular docking verification. The results are shown in Table 2. The affinity between these compounds and the targets was lower than −5.0 kcal/mol, indicating that the core active compounds of rhubarb had a good binding activity with the main target.

**Figure 6:**
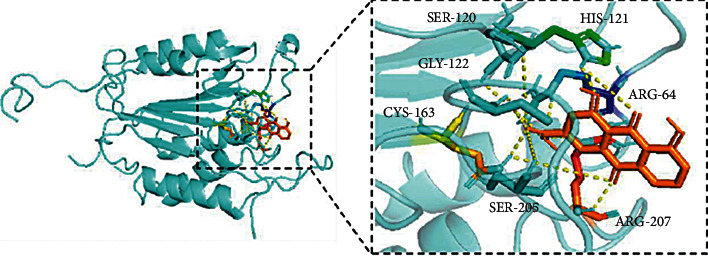
Molecular docking of rhein with CASP3.

The rhein binding with CASP3 showed the highest binding energy (−7.9 kcal/mol).  Figure 6 shows the binding between rhein and CASP3. The amino acid residues SER-120, GLY-122, CYS-163, SER-205, ARG-207, ARG-64, and HIS-121 participated in the hydrophobic interactions.

## 4. Discussion

As a kind of traditional Chinese medicine, rhubarb has a wide range of pharmacological effects and has many functions such as antibacterial and anti-inflammatory. There have been increasing reports on the treatment of tumors using rhubarb in recent years, but its mechanism of action is not yet clear. Network pharmacology is consistent with the thinking of traditional Chinese medicine, which helps fully explain the antitumor mechanism of rhubarb. In this paper, a total of 10 active ingredients with OB ≥ 30% and DL ≥ 0.18 were screened in the TCMSP database. Among them, there are increasing reports about emodin, rhein, aloe-emodin, and so on. Many studies have found that they can exert their antitumor effects through a variety of ways, such as inhibiting tumor cell proliferation, promoting apoptosis, inhibiting invasion and metastasis, and combining radiotherapy and chemotherapy drugs to attenuate and increase efficacy.

The component-target network reflects the antitumor features of rhubarb with multiple components and multiple targets. The PPI network shows that there were interactions between the targets of rhubarb. Among them, CASP3 (24), JUN (24), MYC (23), TNF (20), and PTGS2 (19) are the top 5 nodes with the highest degree value, and it was speculated that they may be the main targets of rhubarb's antitumor effects. CASP3 occupies an essential position in cell apoptosis. Once activated, the cell will die irreversibly. Li et al. reported that aloe-emodin treatment could inhibit the cell viability of SCC15 cells, and the potential mechanism of inhibition might be through the induction of apoptosis by regulation of the expression levels of caspase-9 and caspase-3 [26]. Li et al. reported that rhein inhibits SGC-7901 proliferation by inducing apoptosis, and this antitumor effect of rhein is mediated in part by an intrinsic mitochondrial pathway and the expression of caspase-3 significantly increased [27]. Du et al. studied the effect of rhein on liver cancer HepG2 cells, and they found that it can directly act on mitochondria, causing mitochondrial permeability conversion, and Ca2^+^ and cytochrome C released into the cytoplasm, triggering the activation of caspase 3 and subsequent apoptosis [28]. JUN (transcription factor AP-1) is a heterodimer composed of Fos protein and Jun protein encoded by proto-oncogene. Its activation is involved in the process of cell proliferation, differentiation, transformation, and apoptosis. MYC proto-oncogene protein is an important transcription factor that regulates the expression of many genes and has an impact on cell growth, differentiation, and apoptosis. TNF, tumor necrosis factor, can induce various cytokines and produce an indirect regulation of immune cell activity, enhancing the specific immune protection function. Its function is mainly to regulate immune cells, induce cell apoptosis, prevent tumorigenesis and virus replication, etc. PTGS2 is a key enzyme in prostaglandin biosynthesis. Its product, prostaglandin, is related to inflammation and mitogenesis. It has biological activities such as inhibiting cell apoptosis, promoting cell proliferation, and promoting angiogenesis, indicating that PTGS2 plays an essential role in tumor occurrence and development [29].

From the analysis of pathways, the antitumor pathways of rhubarb mainly involve cancer pathway, calcium signaling pathway, apoptosis, small-cell lung cancer pathway, p53 signaling pathway, and tumor necrosis factor TNF signaling pathway. The calcium signal pathway is widely involved in the entire physiological process of the normal cell cycle, such as cell growth, proliferation, differentiation, etc. Increasing studies have found that the remodeling or abnormality of the calcium signal pathway is an important relevant feature of cancer cells. P53 is a tumor suppressor protein, which has a regulatory effect on the expression of various genes, including apoptosis and inhibition of cell cycle progression. The pathways mentioned above are closely related to the antitumor effect, indicating that rhubarb has a good antitumor effect.

The active molecules of rhubarb had a good binding activity with key target proteins, indicating that the molecular docking results were consistent with the results of network pharmacology, which further proved the reliability of network pharmacology prediction targets.

## 5. Conclusions

Traditional Chinese medicine has been passed down for thousands of years in China. This study explored the role of rhubarb in the treatment of tumors through network pharmacology and molecular docking. After screening and analysis, 10 active ingredients and 58 antitumor effect prediction targets in rhubarb were obtained, involving multiple pathways and multiple targets. This study provides clues for understanding the molecular mechanisms of potential targets of rhubarb in the treatment of cancer and provides a reference for further experimental study.

There are some defects in the present work. This study lacks experimental verification, which will be further validated in future studies.

## Figures and Tables

**Table 1:** Potential effective ingredients of Chinese rhubarb.

Number	Chemical compound	OB/%	DL
1	Eupatin	50.80	0.41
2	Physciondiglucoside	41.65	0.63
3	Rhein	47.07	0.28
4	Torachrysone-8-O-beta-D-(6′-oxayl)-glucoside	43.02	0.74
5	Toralactone	46.46	0.24
6	Emodin-1-O-beta-D-glucopyranoside	44.81	0.80
7	Daucosterol_qt	35.89	0.70
8	Beta-sitosterol	36.91	0.75
9	Aloe-emodin	83.38	0.24
10	(−)-Catechin	49.68	0.24

**Table 2:** Molecular docking scores of major active compound-main target molecular docking.

Number	Compound	Affinity value (kcal/mol)	
		CASP3	JUN
1	Eupatin	−7.1	−5.8
3	Rhein	−7.9	−6.3
5	Toralactone	−7.0	−5.9
8	Beta-sitosterol	−7.8	−6.6
9	Aloe-emodin	−7.3	−6.1
10	(−)-Catechin	−7.0	−5.7

## Data Availability

The data used to support the findings of this study are available from the corresponding author upon request. The authors got the composition of rhubarb and its potential target from the TCMSP database (http://tcmspw.com/tcmspsearch.php). They also got the potential target of cancer according to the OMIM database (http://www.omim.org/) and GeneCards database (http://www.genecards.org) and, subsequently, PPI analysis (STRING Database, https://string-db.org/), KEGG pathways analysis, and GO biological processes (David Database, https://david.ncifcrf.gov/).

## Supplementary Materials

Supplementary Materials Supplemental Material 1: component and targets of rhubarb. Supplemental Material 2: tumor-related genes. Supplemental Material 3: the list of 58 antitumor targets of rhubarb.
